# Supplementary material for: Patterns of Intron Gain and Loss in Fungi
Source: PLoS Biol. 2004 Nov 30;2(12):e422. doi: 10.1371/journal.pbio.0020422 (PMC532390; doi:10.1371/journal.pbio.0020422)
Supplement: Table S1 — Also available at http://genes.mit.edu/NielsenEtAl/. (4.3 MB ZIP). [file pbio.0020422.st001.zip › NielsenEtAl/html/654.html]

AN5132.1.NCU03561.1.MG03224.1.FG00646.1


```
 CLUSTAL W (1.82) Multiple Sequence Alignments - Introns Inserted


Sequence 1: NCU03561.1	304 aa
Sequence 2: MG03224.1	304 aa
Sequence 3: FG00646.1	301 aa
Sequence 4: AN5132.1	324 aa
Alignment Length: 328 aa
Number Identitical Residues: 222 aa
Alignment Score (without introns) 9190


MG03224.1 	MSPPIAAGR-----EKESNLARLLGSG~SAGISELMLFHP0VDTIAKRLMSNETK0VQNI
NCU03561.1	MSP-VAAGRS----EKESNLARLLGSG1SAGIAELAIFHP0VDTIAKRLMSNETK0ISNT
FG00646.1 	MSP---PGGS----ERESGLARVLGSG1SAGIAELAVFHP0VDTIAKRLMSNQSR0ISGS
AN5132.1  	MSPAAAHGSASKDVKKESATARLLGSG1SAGIAELLVFHP0VDTTAKRLMSNQTR0ITSA
          	***. : * ::.. ::**  **:**** ****:** :*** *** *******::: : . 

MG03224.1 	TQLNSVIFKEKATAPVSKKFFSLFPGLGYAAGYKV------------------LQRIYKY
NCU03561.1	AELNKVIFKNKAEAPFGRKFVSLFPGLGYAAGYKV------------------LQRIYKY
FG00646.1 	SQLNQVIFRDTASATVGKKFVSLFPGLGYAAGYKV------------------LQRIYKY
AN5132.1  	SEFNQVVFKEYANAPLARKFTSLFPGLGYAAGYKVGRTKRWLWLPLAHKTPQVLQRIYKY
          	:::*.*:*:: * *...:** **************. :.     . : .:.. *******

MG03224.1 	GGQPIARDALTANFGKDFENAFGKKTGKAILHSTAGS~LIGIGEIVLLPLDVLKIKRQTN
NCU03561.1	GGQPVARDFLGTHYGKDFENAFGKKTGKAIMHSTAGS~LIGIGEIVLLPLDVLKIKRQTN
FG00646.1 	GGQPVARDFLAKNYGSDFENAFGKKTGKAIMHSTAGS2LIGIGEIVLLPLDVLKIKRQTN
AN5132.1  	GGQPFARDYLAKHHGADFDKAFGKGTGKAIMHATAGS~LIGIGEIVLLPLDVLKIKRQTN
          	****.*** *  :.* **::**** *****:*:**** **********************

MG03224.1 	PEAFRGRGVLAIVKDEGFG-LYRGWGWTAARNAPGSFA0LFGGSAFAKEFLFKLEDYNKA
NCU03561.1	PEAFKGRGVFKIIKDEGFGNLYRGWGWTAARNAPGSFA~LFGGSAFAKEFLFGLNDYNKA
FG00646.1 	PEAFRGRGVLKIVADEGFG-LYRGWGWTAARNAPGSFA0LFGGSAFAKEYLFHLEDYNKA
AN5132.1  	PEAFRGRGLFKIISDEGMG-LYRGAGWTAARNAPGSFA0LFGGSAFAKEYIYSLQDYNKA
          	****:***:: *: ***:* **** ************* **********::: *:*****

MG03224.1 	TWFQNFVASIAGSSASLVVSAPLDVIKTRIQNRNFENPESGFRILSNMAKNEGFSSFFKG
NCU03561.1	TWFQNFVASIAGASASLVVSAPLDVIKTRIQNRNFDNPESGFRILTNMAKNEGAGAFFKG
FG00646.1 	SWFQNFVASIAGASASLVVSAPLDVIKTRIQNRNFENPESGFRILSNMAKNEGAGAFFKG
AN5132.1  	SWAQNFVASVCGASASLIVSAPLDVIKTRIQNRNFENPESGFRIVSNMMKNEGPTAFFKG
          	:* ******:.*:****:*****************:********::** ****  :****

MG03224.1 	LVPK0LLMTGPKLTFSFWLAQTLIPAFDNILARS
NCU03561.1	LVPK~LLMTGPKLVFSFWLAQTLIPAFDIAFRK-
FG00646.1 	LVPK0LLMTGPKLVFSFWLAQTLIPAFDIAFKK-
AN5132.1  	LTPK~LLMTGPKLVFSFWLAQTLIPAFGQIV---
          	*.** ********.*************.  .
```
